# Effect of a polymerization inhibitor on the chemomechanical properties and consistency of experimental resin composites

**DOI:** 10.1590/0103-6440202204242

**Published:** 2022-06-24

**Authors:** Caio Vinícius Signorelli Grohmann, Mário Alexandre Coelho Sinhoreti, Eveline Freitas Soares, Robson Ferraz de Oliveira, Eduardo José de Carvalho Souza-Júnior, Saulo Geraldeli

**Affiliations:** 1 Department of Restorative Dentistry, Dental Materials Division, Piracicaba Dental School, University of Campinas, Piracicaba, SP, Brazil.; 2 Division of Biomedical Materials, School of Dental Medicine, East Carolina University, Greenville, NC, USA.

**Keywords:** Butylated hydroxytoluene, composite resin, mechanical properties, degree of conversion

## Abstract

This study investigated the effect of butylated hydroxytoluene (BHT) inhibitor on degree of conversion (DC), flexural strength (FS), flexural modulus (FM), Knoop microhardness (KH), microhardness reduction (HR), and consistency of experimental resin composites at different BHT concentrations: C0 (control-0%); C0.01 (0.01%); C0.025 (0.025%); C0.05 (0.05%); C0.1 (0.1%); and C0.5 (0.5%). For the consistency, the composites were tested immediately after being exposed to a dental chair headlight (0, 20, 40 and 60 s). Data concerning DC, FS, FM, KH, and HR were submitted to one-way ANOVA, while the consistency data was submitted to 2-way ANOVA; mean values were then compared (Tukey’s test; α=0.05). The KH, FS and FM analyses showed no significant difference among the composites tested. For DC, C0 showed the highest mean value (74.2%) and differed only from C0.5 (67.2%). For HR, C0.5 showed the lowest mean (13.09%) value and differed from C0 (26.4%) and C0.01 (24.87). The consistency analysis showed no difference among C0.05, C0.1 and C0.5, considering 0 and 20 s of light exposure, while C0 (14.07 mm), C0.01 (13.97 mm), and C0.025 (14.18 mm) showed higher mean values at 0 s when compared to 20 s (12.67, 12.77 and 13.05 mm, respectivelly). Polymerization occurred within 40 s of light exposure for C0, C0.01, C0.025, and C0.05 and within 60 s for C0.1. In conclusion, the BHT concentrations had no significant influence on FS, FM and KH. The higher the BHT concentration, the longer was its handling time under light, with a significant improvement in the HR, but a decrease in DC. Therefore, BHT at 0.1% showed the best outcomes concerning all the BHT concentrations tested.

## Resumo

O objetivo foi investigar o efeito do inibidor de polimerização BHT no grau de conversão (GC), resistência à flexão (RF), módulo de flexão (MF), microdureza Knoop (KH) e redução da microdureza (RKH), e consistência de um compósito experimental contendo diferentes concentrações de BHT (% em peso): C0 - controle (0%); C0,01 (0,01%); C0,025 (0,025%); C0,05 (0,05%); C0,1 (0,1%); e C0,5 (0,5%). Para o teste de consistência, os compósitos foram testados imediatamente após serem expostos à luz de um refletor odontológico por 0, 20, 40 e 60 s. Os dados relativos a GC, RF, MF, KH e RKH foram submetidos a *one-way* ANOVA, enquanto os dados de consistência foram submetidos a *two-way* ANOVA; os valores médios foram comparados (teste de Tukey; α = 0,05). As análises de KH, RF e MF não mostraram diferença significativa entre os compósitos testados. Para GC, C0 apresentou o maior valor médio e diferiu apenas de C0,5. Para a RKH, C0,5 apresentou o menor valor médio e diferiu de C0 e C0,01. A análise de consistência não mostrou diferença entre C0,05, C0,1 e C0,5, considerando-se 0 e 20 s de exposição à luz, enquanto C0, C0,01 e C0,025 apresentaram maiores valores médios a 0 s quando comparados a 20 s. A polimerização ocorreu dentro de 40 s de exposição à luz para C0, C0.01, C0.025 e C0.05 e dentro de 60 s para C0.1. Em conclusão, as concentrações de BHT não tiveram influência significativa sobre RF, MF e KH. Quanto maior a concentração de BHT, maior o tempo de manuseio sob luz, com melhora significativa da RKH, mas diminuição da GC. Portanto, o compósito contendo 0,1 % de BHT apresentou os melhores resultados entre as demais concentrações testadas.

## Introduction

Resin composite is the most commonly used material to restore anterior and posterior teeth, due to its aesthetic and easy-to-handle characteristics ^1^ ^,^ ^2^. It is photoactivated by high intensity light, which converts monomers into polymers, transforming the viscous mass into a solid material (3;4;5). During light irradiation, free radicals in the composite trigger the monomer’s polymerization and a molecule cross-linking reaction ^6^ ^,^ ^7^.

The polymerization technology for current composites depends on photoinitiator and co-initiator systems, suitable for the absorption of specific wavelength irradiation light, and capable of converting a monomer into a cross-linking network ^8^ ^,^ ^9^. Technically, polymerization starts when the resin composite is exposed to light, either from a reflector or naturally from the environment. However, the speed of the chemical reaction would be slower when compared to the speed of the chemical reaction activated by the light emitted from a dental light-curing unit. Nevertheless, this exposure may impair the mechanical properties of the material and hinder its proper manipulation due to a working time reduction ^10^ ^,^ ^11^ ^,^ ^12^.

Polymerization inhibitors are antioxidant molecules added to monomeric bases to inhibit premature and spontaneous polymerization. Hydroquinone (HQ), monomethyl ether hydroquinone (MEHQ), and butylated hydroxytoluene (BHT) are commonly found in formulations of dental resin composites. Although, BHT is the most used polymerization inhibitor in commercial composites ^13^ ^,^ ^14^. BHT concentration in resin composites is around 0.01%/wt. ^15^ ^,^ ^16^. Inhibitors aim to prevent polymerization of composites during light (artificial or natural) exposure, because they stop every free radical, and the polymerization is completely halted until they are consumed. When the inhibitor has been consumed, the polymerization proceeds at the same rate as in the absence of inhibitor ^16^. To be effective, the inhibitors have to react with photo-initiator free radicals faster than such free radicals react with the monomers ^17^ ^,^ ^18^.

The degree of conversion (DC) tends to decrease as the inhibitor concentration increases ^19^. However, the effect of the inhibitors on the mechanical properties of resin composites remains little studied. Al-Shammari ^10^ reported that some fine-tuning in the concentration of the inhibitor can effectively prevent early polymerization of the material without compromising its degree of conversion. The author added that an ideal inhibitor concentration leads to a suitable rate of polymerization, without impairing the mechanical properties of the material ^10^.

Some studies on inhibitor concentration in resin composites have aimed at controlling and minimizing their polymerization shrinkage stress (10, 20). Frequently, professionals have challenges when applying, adapting and modeling the resin composite into the tooth preparation due to early polymerization caused by the dental chair headlight, leading professionals to either switch off the light or use orange colored light ^16^.

The aim of the present study was to investigate the effect of different concentrations of BHT on DC, flexural strength (FS), flexural modulus (FM), Knoop microhardness (KH), microhardness reduction (HR), and the consistency of six experimental resin composites. The hypotheses tested were that ^1^ the BHT concentration would have no effect on the DC and mechanical properties of the composites tested, and ^2^ that higher BHT concentrations would lead to lower consistency of the material after dental chair headlight exposure.

## Material and methods

### Resin composite preparation

Six experimental resin composites were prepared using a centrifugal mixing device (SpeedMixer, DAC 150.1 FVZ- K, Hauschild Engineering, Germany). The resin matrix for all formulations was made of 30 wt.% bisphenol glycidyl methacrylate (BisGMA - Sigma-Aldrich Inc, St Louis, MO, USA), 30 wt.% urethane dimethacrylate (UDMA - Sigma-Aldrich Inc., USA), 30 wt.% bisphenol ethoxylate dimethacrylate (BisEMA - Sigma-Aldrich Inc., USA), and 10 wt.% triethyleneglycol dimethacrylate (TEGDMA - Sigma-Aldrich Inc., USA), including a photoinitiator - 0.25 wt.% camphorquinone (CQ - Sigma-Aldrich Inc., USA) - and a co-initiator - 0.5 wt.% N,N-dimethyl-p-aminobenzoic acid ethylester (DABE - Sigma-Aldrich Inc., USA).

The resin matrix was loaded with 75 wt.% silanized filler, 20 wt.% of which was 0.05 μm fumed silica (Nippon Aerosil Co. Ltd., Yokkaichi, Tokyo, Japan) and 80 wt.% of 0.7 μm Ba-Al-silicate glass (Esstech Inc., Essington, PA, USA). The only difference among the experimental resin composites was the different weight concentrations of the polymerization inhibitor - butylated hydroxytoluene (BHT - Sigma-Aldrich Inc., USA): 0 - control (C0); 0.01 wt.% (C.01); 0.025 wt.% (C.025); 0.05 wt.% (C.05); 0.1 wt.% (C.1); and 0.5 wt.% (C.5).

### Consistency (C)

The consistency test was carried out according to specification No. 8 of the American Dental Association. The idea is that the more the resin composite maintained its initial consistency when exposed to dental chair headlight, the longer the working time available for the dentist to adapt and shape the material into the preparation. Each resin composite was hemi-spherically shaped into rubber molds (0.5 mL). The specimens (n=5) were demolded, placed onto a glass plate, and exposed (except for the control) to the dental chair headlight (quartz-tungsten-halogen lamp, 150 W, Osram, Berlin, Germany) for 20, 40 and 60 s, at a distance of 50 cm. After light exposure, another 20 grams glass plate plus a load weight of 100 grams were placed over the resin composite for 30 seconds to fabricate the composite discs specimens.

The load weight was removed, and the disc-shaped specimen was photoactivated for 20 s (Radii Cal, SDI, Victoria, Australia - 800 mW/cm^2^). The specimens’ minor and major diameters were measured (mm) using a digital caliper (Mitutoyo Corporation, Tokyo, Japan). The mean values of the minor and major measurements were obtained for each specimen separately, as well as for the five specimen combined in each group.

### Degree of conversion (DC)

For the DC analysis, 5 specimens (7 mm in diameter x 1.5 mm thick) for each group were fabricated in rubber molds and photoactivated (Radii Cal, SDI, Australia - 800 mW/cm^2^) for 25 s. The total energy dose was standardized at 20 J/cm^2^. The specimens were then removed from the molds and dry-stored in light-proof containers at 37 ºC, for 24 h. The DC of the top surface of each specimen was measured using the Fourier transformed infrared spectroscopy with attenuated total reflectance (FTIR/ATR - Spectrum 100, PerkinElmer, Shelton, CA, USA).

The absorption spectra of both polymerized and non-polymerized experimental resin composites were recorded within a range of 1500 to 1800 cm^-1^ with an acquisition time of 10 s with the recorded spectra averaged over three successive measurements in distinct points. DC was calculated by estimating the changes in the peak height ratio (R) of the absorbance intensities of the aliphatic C=C peak at 1638 cm^-1^ and that of an internal standard peak of the aromatic C=C at 1608 cm^-1^ during polymerization, using the following equation:

DC (%)= 100 ×[1 - (R polymerized )/(R non-polymerized)]

### Flexural strength (FS) and flexural modulus (FM)

The bar-shaped specimens (n=10; 25 mm long x 2 mm thick x 2 mm wide) were used for the three-point bending flexural test, accordingly to ISO 4049. The three-point bending test was carried out using a universal testing machine (Instron, Canton, USA ) at a crosshead speed of 0.5 mm/min with supports set at 20 mm apart. The maximum load for each specimen was recorded at fracture and the FS (σ) was calculated using the following equation: σ = 3Fl/2bh², where F indicates the maximum load (N) exerted on the specimens; l, the distance (mm) between the supports; b, the width (mm) and h, the height (mm) of the specimens measured immediately prior to testing. The FM (E) was calculated using the following equation: E = ld³/4wh³D x 10^-3^, where l indicates the maximum fracture load (N); d, the distance (mm) between the supports; w, the width (mm); h, the height (mm); and D, the deflection (mm).

### Knoop microhardness (KH)

Specimens (n=5) of each resin composite were fabricated in a rubber mold with a central circular orifice, into which the composite was inserted in one single increment. The circular specimens (7 mm in diameter and 1.5 mm in thickness) were then photoactivated for 20 s (Radii-cal LED, 800 mW/cm²; SDI, Australia) and stored for 24 h at 37 °C, in dry conditions and protected from any source of light.

The specimen surfaces that were exposed to curing light were submitted to the Knoop microhardness test using in a micro-durometer (HMV-2000, Shimadzu, Tokyo, Japan), at a load of 50 g for 15 s (five indentations were made on each specimen). The values (in micrometers) were converted into Knoop microhardness values (KHN) using the micro-durometer software.

### Microhardness reduction (HR)

After being tested for KH, the disk-shaped specimens (n=5) were stored in 100% ethanol (Sigma-Aldrich Inc, USA) for 24 h and then submitted to the chemical softening test. Afterwards, the specimens were tested for KH again - five readings for each specimen, considering the same surface previously tested - were done to verify the HR (values were expressed as a percentage). The average of the five readings was established as the HR mean value for each specimen.

### Statistical analysis

Power analysis was conducted to determine sample size for each experiment to provide a power of at least 0.8 at a significance level of 0.5 ((=0.2). The C data were submitted to 2-way ANOVA and those concerning DC, FS, FM, KH, and HR to one-way ANOVA. The mean values were compared using Tukey’s test. The significance level of α=0.05 was established for all tests.

## Results

The consistency (C) results are shown in Table 1. No statistical difference was observed for the control (no light exposure). For the 20 s light exposure groups, C0.05, C0.1 and C0.5 showed the highest mean values and were statistically different from C0, C0.01 and C0.025 (p=0.0001). For the 40 s groups, only C0.1 and C0.5 showed consistency values, with C0.5 showing a mean value statistically higher than that of C0.1 (p=0.0001). At 60 s, only C0.5 showed consistency values.

**Table 1:** Consistency diameter’s mean values (standard deviation), in mm, for the experimental resin composites tested without light exposure (control) and under different times of light exposure (20, 40 or 60 seconds).

Composite	Time (s)			
	0 (control)	20	40	60
C0	14.07 (0.45) a, A	12.67 (1.16) b, B	0.0 (0.0) c, C	0.0 (0.0) b, C
C0.01	13.97 (0.16) a, A	12.77 (0.48) b, B	0.0 (0.0) c, C	0.0 (0.0) b, C
C0.025	14.18 (0.65) a, A	13.05 (0.62) b, B	0.0 (0.0) c, C	0.0 (0.0) b, C
C0.05	14.02 (0.52) a, A	13.78 (0.47) a, A	0.0 (0.0) c, B	0.0 (0.0) b, B
C0.1	14.21 (0.49) a, A	13.61 (0.66) a, A	8.42 (0.38) b, B	0.0 (0.0) b, C
C0.5	14.32 (0.25) a, A	14.23 (0.19) a, A	13.49 (0.44) a, B	8.96 (0.39) a, C

Mean values followed by different lowercase letters in columns and capital letters in rows indicate statistical difference (p<0.05; Tukey’s test).

Statistical differences were observed among the exposure times for all experimental composites (Table 1), except for C0.05, C0.1 and C0.5, considering 0 (control) and 20 s. C0, C0.01, and C0.025 showed mean values significantly higher for the control (no exposure time) when compared with 20 s (p=0.0001). The resin composites C0, C0.01, C0.025, and C0.05 polymerized under dental chair headlight from 40 s and C0.1 at 60 s. Thus, only under C0.5 could all the exposure times be compared. The consistency mean values for C0.1 and C0.5 increased statistically (p=0.0001) as the exposure time increased.

Table 2 shows the mean values for FS, FM, and DC. No significant difference was observed among the composites concerning FS (p=0.41797) and FM (p=0.15213). Regarding DC, C0 showed the highest mean values and statistically differed (p=0.03760) from C0.5. No significant difference was observed among the other groups - C0.01, C0.025, C0.05 and C0.1.

**Table 2:** Mean values (standard deviation) for FS, FM, and DC of experimental resin composites.

Composite	FS (MPa)	FM (GPa)	DC (%)
C0	96.85 (12.82) a	1.65 (0.1) a	74.2 (4.6) a
C0.01	93.27 (10.19) a	1.42 (0.27) a	72.1 (3.4) ab
C0.025	97.74 (15.96) a	1.63 (0.2) a	73.7 (2.2) ab
C0.05	94.89 (10.37) a	1.6 (0.2) a	72.4 (3.5) ab
C0.1	93.42 (6.7) a	1.52 (0.21) a	73.3 (4.7) ab
C0.5	94.77 (15.09) a	1.77 (0.16) a	67.2 (3.1) b

Mean values followed by different lowercase letters in columns are statistically different (p<0.05; Tukey’s test).

Table 3 shows the mean values of KH before (no-ethanol) and after chemical softening (ethanol) and HR. No statistical difference was observed among the experimental resin composites concerning KH. After chemical softening, C0.5 showed the lowest HR value and differed from C0 and C0.01 (p=0.0001). No significant difference was observed among the other resin composites (C0.025, C0.05, and C0.1).

**Table:** 

Composite	no-ethanol (KHN)	etanol (KHN)	HR (%)
C0	57.34 (3.75) a	42.21	26.40 (2.0) a
C0.01	56.04 (4.11) a	42.11	24.87 (2.2) a
C0.025	55.25 (2.93) a	44.32	19.81 (2.5) ab
C0.05	55.39 (3.21) a	45.50	17.85 (1.2) ab
C0.1	56.42 (1.82) a	45.02	20.24 (1.7) ab
C0.5	55.46 (3.09) a	48.25	13.09 (2.5) b

Mean values followed by different lowercase letters in columns are statistically different (p<0.05; Tukey’s test).

## Discussion

The first hypothesis, which stated that the concentration of BHT would not influence the degree of conversion and mechanical properties of the experimental resin composites, was rejected because BHT at different concentrations had a significant impact on the DC and HR results. BHT at the highest concentration (C0.5) decreased the DC and HR values significantly when compared to the control (C0). In contrast, the second hypothesis in the present study was not rejected since the most favorable BHT concentration was 0.5%, showing satisfactory consistency, even after 60 s of light exposure. C0.1 (0.1%) also showed satisfactory chemical and mechanical properties and lower consistency up to 40 s of light exposure

BHT at a high concentration might lead to a very slow chemical reaction during photocuring, resulting in a decrease in DC. This might be due to less available free radicals that are competitively quenched by the inhibitor ^16^. The optimal BHT concentration should be defined to ensure adequate polymerization of the resin composite, as well as longer shelf life and handling time. The BHT concentration varies among commercial resin composites ^21^ and its effects on the polymerized polymeric structure remain unclear.

According to Braga and Ferracane ^19^, the higher the inhibitor’s concentration, the lower the curing rate and the shrinkage stress. Although statistically insignificant, the DC decreased as the inhibitor’s concentration increased; however, the effects of the inhibitor’s concentrations on mechanical properties were not evaluated in their study. Al-Shamari ^10^ showed that the inhibitor at an optimal concentration leads to polymerization at a rate slow enough to reduce the shrinkage stress, with a DC that does not impair the mechanical properties of the resin composite.

The highest DC mean values were obtained for the inhibitor’s concentration ranging from 0 to 0.1 %, with no impact on FS and FM. This could be explained by the cyclization process in which both ends of the polymer chain react with each other, terminating the chain growth. According to Elliott et al. ^22^, such cyclization produces an isolated “microgel,” having no cross-link with the surrounding resin chains. This process results in a higher local DC associated with a lower cross-link density. One of the limitations is that the present study included no fractographic analysis of the FM test samples; such analysis could provide a better understanding of the influence of the BHT concentration on the cyclization process of the polymeric chains in formation.

After chemical softening, C0 and C0.01 (lowest BHT concentration) showed HR mean values statistically higher than C0.5 (highest BHT concentration). This might be due to a higher BHT concentration in C0.5 - when compared to C0 and C0.01 - leading to a higher cross-link density. It has been observed that cross-linked dimethacrylate networks swell when exposed to solvents ^23^. This occurs because the forces of attraction between the polymer chains are exceeded by the forces of attraction between solvent molecules and components of the chains, breaking the cross-links among the polymer chains ^23^ ^,^ ^24^ ^,^ ^25^. Absence of BHT, or at low concentrations, might result in the cyclization of the polymer chains, reducing its cross-link density.

The findings concerning DC and HR had no impact on the FS, FM and HK tests and are in disaccord with those reported by Al-Shamari ^10^ and Ferracane & Greener ^24^. This disparity might be due to the concentrations tested in their studies. In the study of Al-Shammari ^10^, the effect of BHT on FS and FM was observed at concentrations above 1.2%, with a significant decrease when the concentration reached 1.4%. Ferracane & Greener ^24^ reported that FS and FM reduced as the inhibitor concentration increased, a condition attributed to a low cross-link density. In the present study, no reduction in FS, FM, and KH was observed for C0.5 (0.5%), which had both the highest BHT concentration and the lowest HR values after chemical softening. Another limitation of the present study is that no specimen was topographically analyzed after the KH and HR tests to check out for cracks in the indentations produced during these tests; such analysis could provide a better understanding of the effect of the BHT concentration on the hardness and chemical softening of the specimens.

In the present study, the consistency test showed that the higher the BHT concentration of the experiment resin composite, i.e. 0.5%, the greater was the flow of the material, when exposed to the reflector’s light. On the other hand, the resin composites C0, C0.01, C0.025, and C0.05 polymerized under dental chair headlight from 40 s and C0.1 at 60 s, making the consistency measurements in these groups unfeasible. Thus, it is clear how important the BHT concentration is in dental composites in order to achieve adequate working time. Then the most favorable BHT concentration was 0.5% (C0.5), showing satisfactory consistency, even after 60 s of light exposure. The concentration 0.1% (C0.1) also showed satisfactory chemical and mechanical properties and lower consistency up to 40 s of light exposure. Based on these findings, the dental practitioner seems to have a flexible time to handle the material under reflector’s light exposure during the clinical procedures.

Considering the outcomes of the present study, the experimental resin composite with 0.1 wt% of BHT showed optimal mechanical properties and DC, as well as the most satisfactory handling time under the reflector’s light (40 s). However, further studies are needed to investigate whether our findings have any effect on other properties such as color stability, sorption and solubility.
